# Supplementary figures and images for: TMEM120A contains a specific coenzyme A-binding site and might not mediate poking- or stretch-induced channel activities in cells
Source: eLife. 2021 Aug 19;10:e71474. doi: 10.7554/eLife.71474 (PMC8480983; doi:10.7554/eLife.71474)

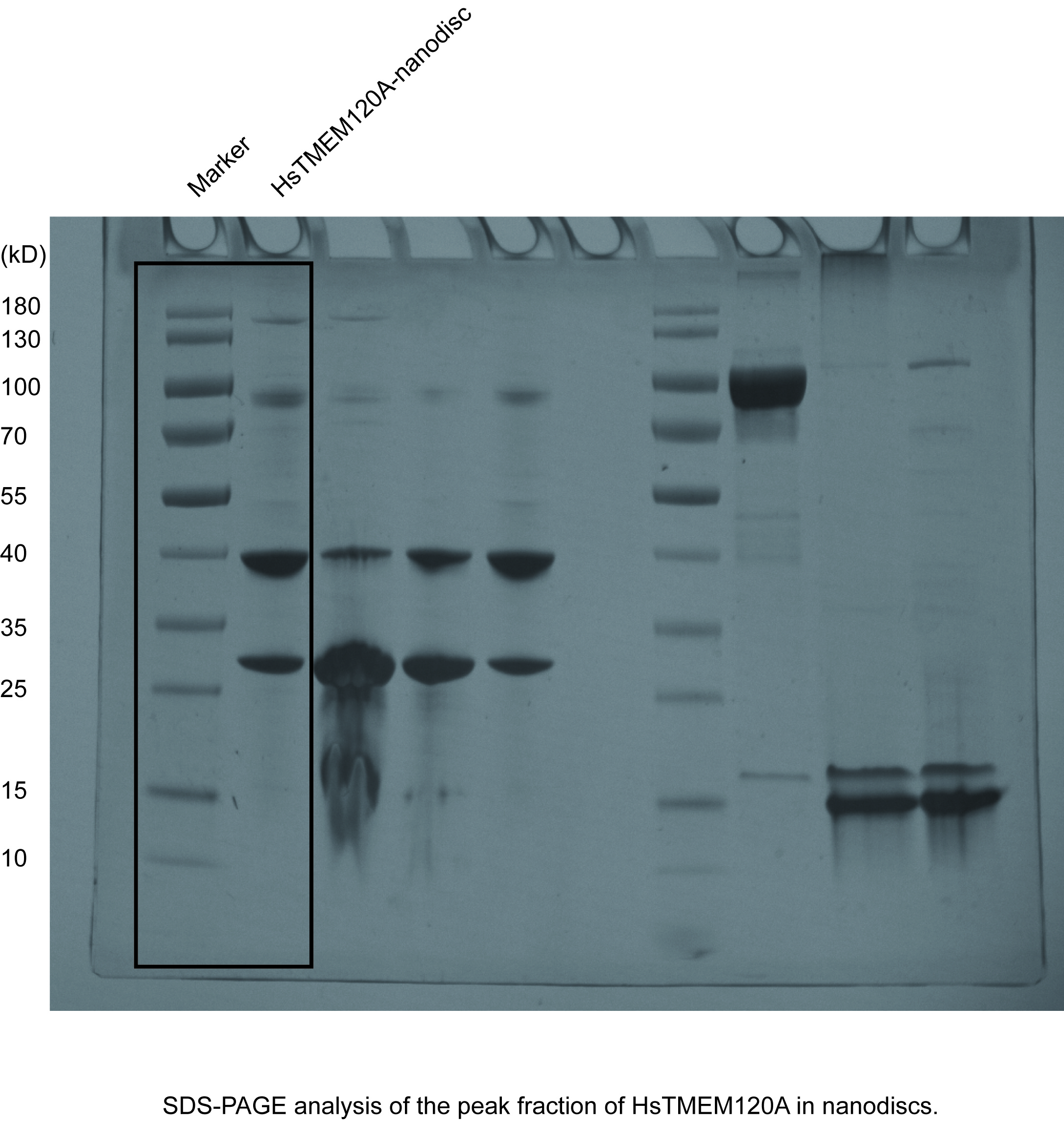

Supplement: Figure 2—figure supplement 1—source data 1. [file elife-71474-fig2-figsupp1-data1.zip › Figure2_Suppl_1A_Source_Data1.jpg]

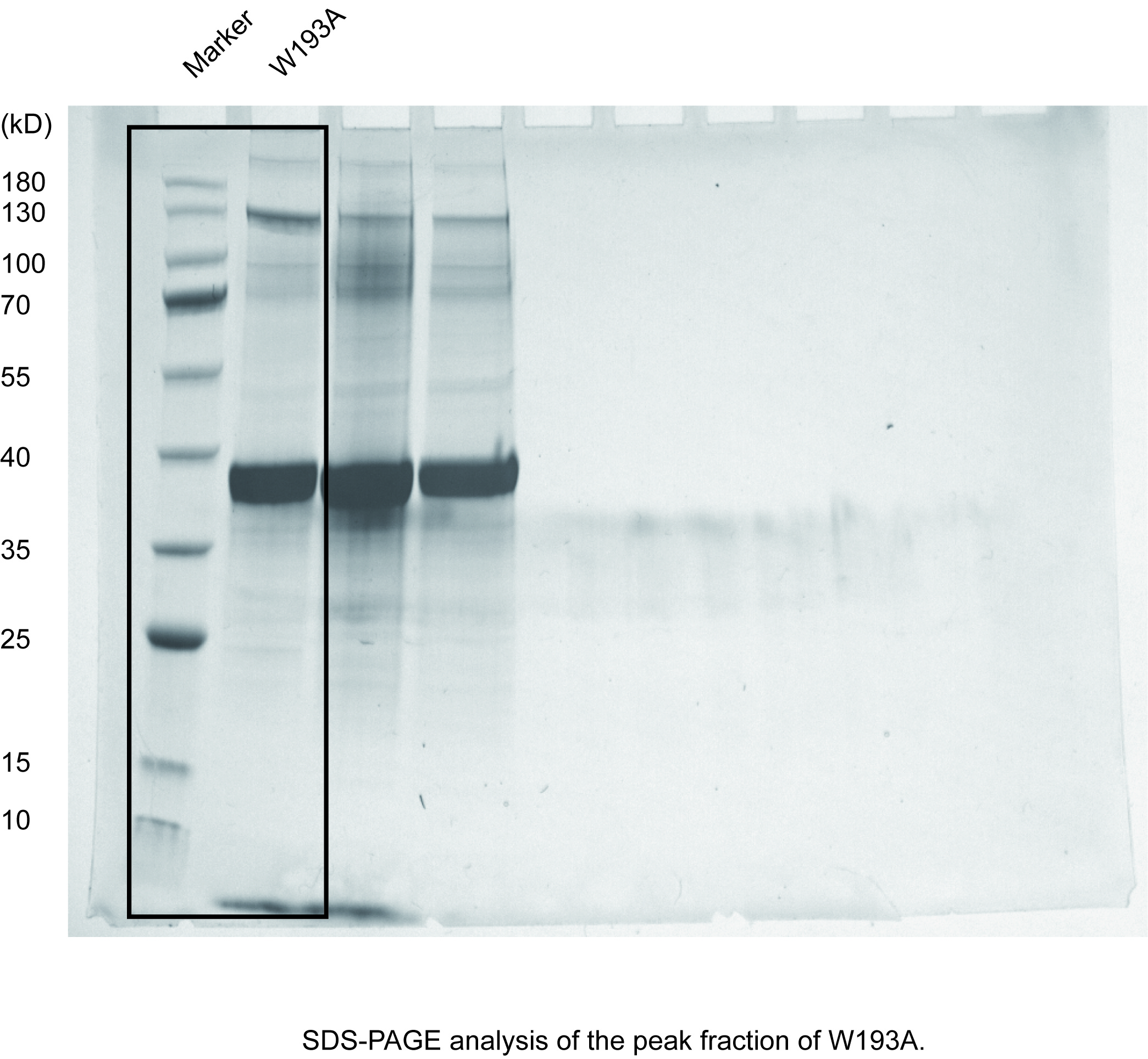

Supplement: Figure 4—figure supplement 4—source data 1. [file elife-71474-fig4-figsupp4-data1.zip › Figure4_Suppl_3A_Source_Data1.jpg]

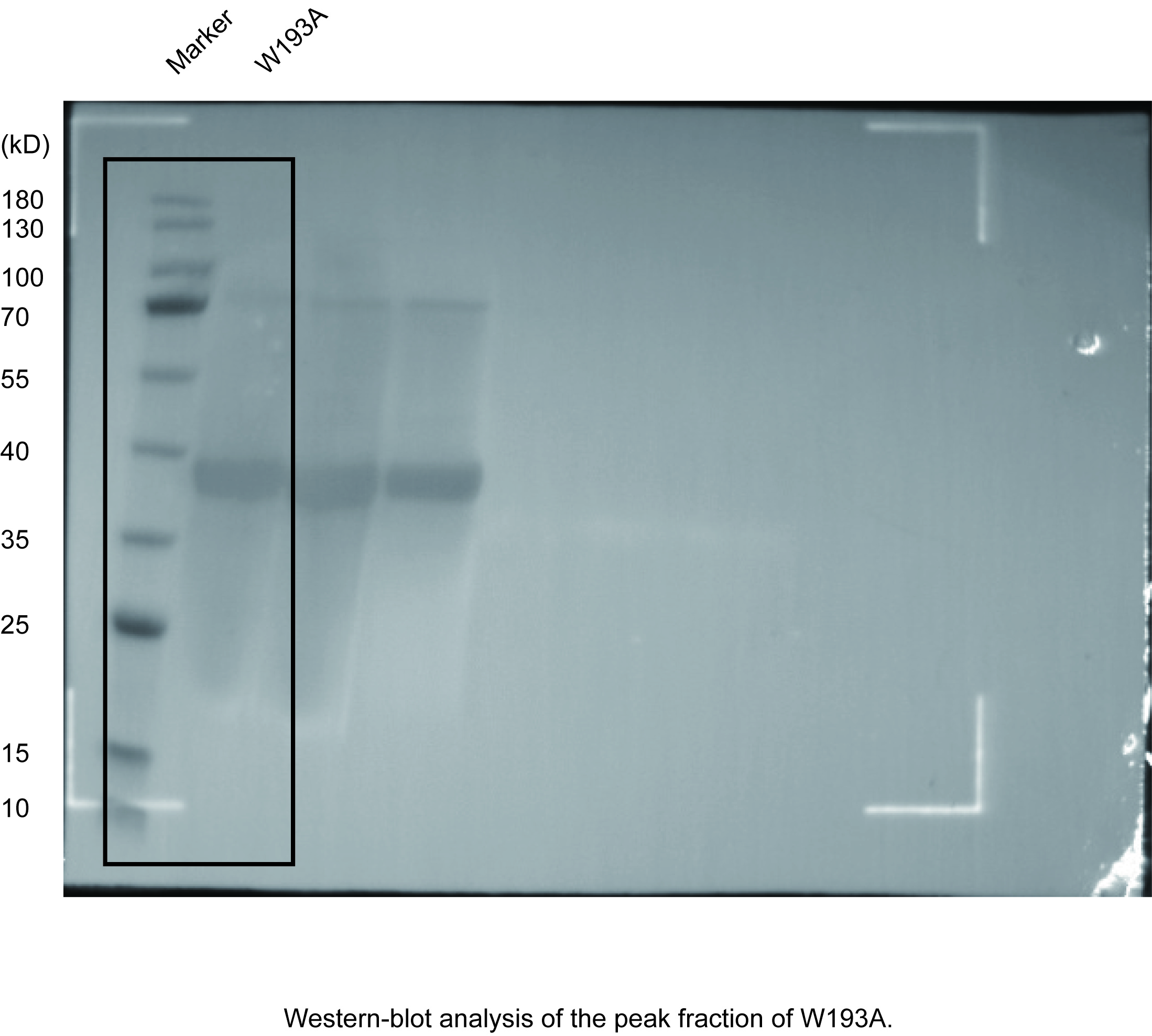

Supplement: Figure 4—figure supplement 4—source data 1. [file elife-71474-fig4-figsupp4-data1.zip › Figure4_Suppl_3A_Source_Data2.jpg]
