# Supplementary material for: TMEM120A contains a specific coenzyme A-binding site and might not mediate poking- or stretch-induced channel activities in cells
Source: eLife. 2021 Aug 19;10:e71474. doi: 10.7554/eLife.71474 (PMC8480983; doi:10.7554/eLife.71474)
Supplement: Supplementary file 1. [file elife-71474-supp1.docx]

**Supplementary Table S1. Cryo-EM data collection and processing, refinement and validation statistics of *Hs*TMEM120A structures.**

|  | *Hs*TMEM120A in nanodiscs | *Hs*TMEM120A in detergent |
| --- | --- | --- |
|  | (EMD-31440) | (EMD-31441) |
|  | (PDB 7F3T) | (PDB 7F3U) |
| **Data collection and processing** |  |  |
| Magnification | 81,000 | 105,000 |

| Voltage (kV) | 300 | 300 |
| --- | --- | --- |
| Electron exposure (e^-^/Å) | 60 | 60 |
| Defocus range (μm) | -1.2 to -1.8 | -1.2 to -1.8 |
| Pixel size (Å) | 1.07 | 0.82 |
| Symmetry imposed | *C*2 | *C*2 |
| Initial particle images (no.) | 6,908,315 | 4,475,146 |
| Final particle images (no.) | 410,963 | 491,986 |
| Map resolution (Å) | 3.69 | 4.0 |
| FSC threshold | 0.143 | 0.143 |
| Map resolution range (Å) | 2.5-6.5 | 2.5-6.5 |
|  |  |  |
| **Refinement** |  |  |
| initial model used (PDB code) | - | - |
| Model resolution (Å) | 3.80 | 4.45 |
| FSC threshold | 0.5 | 0.5 |
| Model resolution range (Å) | - | - |
| Map sharpening *B* factor (Å^2^) | -200 | -277 |
| Model composition |  |  |
| Non-hydrogen atoms | 5632 | 4276 |
| Protein residues | 660 | 656 |
| Water | - | - |
| Ligands | 2 | - |
| *B* factors (Å^2^) |  |  |
| Protein | 52.98 | 120.11 |
| Ligand | 68.41 | - |
| R.m.s. deviations |  |  |
| Bond lengths (Å) | 0.010 | 0.006 |
| Bond angles (°) | 1.221 | 0.865 |
| Validation |  |  |
| MolProbity score | 2.05 | 2.31 |
| Clashscore | 14.73 | 20.67 |

| Poor rotamers (%) | 0.30 | 0 |
| --- | --- | --- |
| Ramachandran plot |  |  |
| Favored (%) | 94.51 | 91.72 |
| Allowed (%) | 5.18 | 8.28 |
| Disallowed (%) | 0.30 | 0 |
