# Supplementary material for: TMEM120A contains a specific coenzyme A-binding site and might not mediate poking- or stretch-induced channel activities in cells
Source: eLife. 2021 Aug 19;10:e71474. doi: 10.7554/eLife.71474 (PMC8480983; doi:10.7554/eLife.71474)
Supplement: Transparent reporting form [file elife-71474-transrepform.docx]

***eLife’s* transparent reporting form**

We encourage authors to provide detailed information *within their submission* to facilitate the interpretation and replication of experiments. Authors can upload supporting documentation to indicate the use of appropriate reporting guidelines for health-related research (see [EQUATOR Network](http://www.equator-network.org/%20)), life science research (see the [BioSharing Information Resource](https://biosharing.org/)), or the [ARRIVE guidelines](http://www.plosbiology.org/article/info:doi/10.1371/journal.pbio.1000412) for reporting work involving animal research. Where applicable, authors should refer to any relevant reporting standards documents in this form.

If you have any questions, please consult our Journal Policies and/or contact us: [editorial@elifesciences.org](mailto:editorial@elifesciences.org).

**Sample-size estimation**

- You should state whether an appropriate sample size was computed when the study was being designed
- You should state the statistical method of sample size computation and any required assumptions
- If no explicit power analysis was used, you should describe how you decided what sample (replicate) size (number) to use

Please outline where this information can be found within the submission (e.g., sections or figure legends), or explain why this information doesn’t apply to your submission:

For patch clamp recordings, sample sizes represent the number of cells tested in whole-cell currents and cell-attached recordings and inside-out patch configuration. The number of cells tested was determined based on the magnitude of the effect observed and the variance among data points. The sizes were chosen based on consistency of data across different conditions and multiple experiments. For electrophysiological experiments on GUV samples, the sample size represents the number of patches tested in the inside-out recordings. The number of patches tested was determined based on the observation of channel activities upon application of negative pressure.

For cryo-EM data collection, the sample size represents the number of different grids loaded with the same batch of protein samples. Two different grids were used for collecting the cryo-EM data of *Hs*TMEM120A nanodisc sample, whereas three different grids were used for collecting the data of *Hs*TMEM120A protein in detergent.

The information about sample size estimation can be found in the Materials and Methods section.

**Replicates**

- You should report how often each experiment was performed
- You should include a definition of biological versus technical replication
- The data obtained should be provided and sufficient information should be provided to indicate the number of independent biological and/or technical replicates
- If you encountered any outliers, you should describe how these were handled
- Criteria for exclusion/inclusion of data should be clearly stated
- High-throughput sequence data should be uploaded before submission, with a private link for reviewers provided (these are available from both GEO and ArrayExpress)

Please outline where this information can be found within the submission (e.g., sections or figure legends), or explain why this information doesn’t apply to your submission:

Electrophysiological experiments were reproduced according to the sample size as indicated in each figure. The recordings and parameters were reproducible across multiple days and transfection batches. Each experiment was performed on cells from at least 3 separate transfections (for positive control in whole-cell patch configuration, 11 separate experiments; in cell-attached configuration, 41 separate experiments; in inside-out patch configuration, 9 separate experiments were performed. for mTMEM120A and hTMEM120A in whole-cell patch configuration, 15 and 10 separate experiments; in cell-attached configuration, 18 and 39 separate experiments; in inside-out patch configuration, 6 and 8 separate experiments were performed. for negative control Vector, in whole-cell patch configuration, 10 separate experiments; in cell-attached configuration, 22 separate experiments; in inside-out patch configuration, 11 separate experiments.) Three separate transfections were performed to acquire TMEM120A only vs vector vs Positive control data.

Electrophysiological experiments to characterize TMEM120A vs vector vs Positive control channels were routinely done by interleaving the cells to be compared to avoid unavoidable differences imposed on the protocol during the course of the experiment such as the time cells were removed from the incubator and fluctuations in room temperature. Electrode wires were chloride coated routinely (2-3 times per week) during the course of these experiments to avoid drift.

Information is provided throughout the manuscript in figure legends indicating the number of independent biological and/or technical replicates.

The cells expressing TMEM120A, positive controls and vector are re-plated as single cell. Cells included in the data set are uniform in size and show similar capacitance.

No data were excluded unless contaminating leak currents obscured the recordings. The acquisition was aborted when the P1-KO-HEK cells revealed a noisy data.

For data shown in Fig. 1E, F, experiments were performed independently by two experimenters (J. J. and W. L.) and similar data were obtained and combined.

For electrophysiological experiments on reconstituted GUV samples, the data shown in Figure1−figure supplement 1A-C were recorded on separate GUV preparations. When the patch exhibited a noisy leaky current, data acquisition was aborted.

**Statistical reporting**

- Statistical analysis methods should be described and justified
- Raw data should be presented in figures whenever informative to do so (typically when N per group is less than 10)
- For each experiment, you should identify the statistical tests used, exact values of N, definitions of center, methods of multiple test correction, and dispersion and precision measures (e.g., mean, median, SD, SEM, confidence intervals; and, for the major substantive results, a measure of effect size (e.g., Pearson's r, Cohen's d)
- Report exact p-values wherever possible alongside the summary statistics and 95% confidence intervals. These should be reported for all key questions and not only when the p-value is less than 0.05.

Please outline where this information can be found within the submission (e.g., sections or figure legends), or explain why this information doesn’t apply to your submission:

For patch clamp recordings, the exact sample size for each experimental group is given as a discrete number and unit of measurement (cells tested from the number of separate cultures/transfections). The number of times each experiment was replicated is included in the figure legend. Since the datapoints fall within a Gaussian distribution, the One-way ANOVA with comparison to the vector was used to compare indicated datasets. Each bar represents mean ± s.e.m., and the recorded cell number is labeled above the bar. P values are given as exact values whenever possible and with confidence intervals noted. For the experiments on GUV samples, the exact sample size (N) for each experimental group/condition is given as a discrete number and unit of measurement (Patches tested from the number of separate GUVs). The number of times for GUV patch experiment was replicated is included in the result section. Each measurement was taken from a single GUV per glass slide.

For quality assessment of the cryo-EM structures, Fourier shell correlation between independently refined half datasets is shown in Figure 2−figure supplement 1D and Figure 5−figure supplement 1D.

(For large datasets, or papers with a very large number of statistical tests, you may upload a single table file with tests, Ns, etc., with reference to sections in the manuscript.)

**Group allocation**

- Indicate how samples were allocated into experimental groups (in the case of clinical studies, please specify allocation to treatment method); if randomization was used, please also state if restricted randomization was applied
- Indicate if masking was used during group allocation, data collection and/or data analysis

Please outline where this information can be found within the submission (e.g., sections or figure legends), or explain why this information doesn’t apply to your submission:

For patch clamp recordings with cells, samples were grouped based on the plasmids transfected into the cells. For recordings on GUV preparations, samples were grouped based on the proteins of interest reconstituted into the GUVs. The investigators were not blinded to group allocation. The information can be found in the Materials and Methods section of the submission.

**Additional data files (“source data”)**

- We encourage you to upload relevant additional data files, such as numerical data that are represented as a graph in a figure, or as a summary table
- Where provided, these should be in the most useful format, and they can be uploaded as “Source data” files linked to a main figure or table
- Include model definition files including the full list of parameters used
- Include code used for data analysis (e.g., R, MatLab)
- Avoid stating that data files are “available upon request”

Please indicate the figures or tables for which source data files have been provided:

The source data files for Figure 1, Figure 1−figure supplement 1, Figure 2−figure supplement 1A, Figure 4D and Figure 4-figure supplement 3 have been provided.

The structural models have been deposited in the Protein Data Bank under accession codes of 7F3T (https://www.rcsb.org/structure/7F3T) for *Hs*TMEM120A-CoASH complex in nanodisc and 7F3U (https://www.rcsb.org/structure/7F3U) for *Hs*TMEM120A in detergent. The cryo-EM density maps of *Hs*TMEM120A-CoASH complex in nanodisc and *Hs*TMEM120A in detergent have been deposited in the Electron Microscopy Data Bank under accession codes of EMD-31440 (https://www.emdataresource.org/EMD-31440) and EMD-31441 (https://www.emdataresource.org/EMD-31441), respectively.
